# Impact of dietary electrolyte balance on performance, inflammation, and gut integrity of lactating sow under heat stress

**DOI:** 10.5713/ab.250610

**Published:** 2025-12-18

**Authors:** Jun Young Mun, Abdolreza Hosseindoust, Priscilla Neves Silvestre, Sang Hun Ha, Habeeb Tajudeen, Jin Soo Kim

**Affiliations:** 1 Department of Animal Industry Convergence, Kangwon National University, Chuncheon, Korea

**Keywords:** Cortisol, Microbiota, Oxidative Stress, Reproductive Performance, Swine, Thermoregulation

## Abstract

**Objective:**

Heat stress adversely affects feed intake, milk production, and overall reproductive performance. One suggested nutritional strategy to mitigate these adverse effects is the optimization of dietary electrolyte balance (dEB) with bicarbonate supplementation, which regulates acid-base homeostasis and thermoregulatory responses. This study aimed to evaluate the effects of different dEB levels on lactating sow reproductive performance during heat stress.

**Methods:**

A total of 40 lactating sows were assigned to four dietary treatments with varying dEB (mEq/kg) levels (230: sodium chloride 0.47%; 250: sodium chloride 0.34%+sodium bicarbonate 0.14%+potassium bicarbonate 0.05%; 270: sodium chloride 0.25%+sodium bicarbonate 0.26%+potassium bicarbonate 0.09%; 290: sodium bicarbonate 0.52%+ potassium bicarbonate 0.13%) to evaluate the effects on reproductive performance, hair cortisol, the acid-base balance, inflammation, gut integrity, behavior, and intestinal microbiota of lactating sows during heat stress.

**Results:**

Increasing dietary dEB levels during heat stress (temperature: 26.0°C–31.1°C; temperature to humidity index: 78–84) linearly increased average daily feed intake of sows during lactation and improved piglet weaning weight. Blood pH decreased linearly with rising dEB levels, while hair cortisol content showed a decreasing trend. interleukin (IL)-1β tended to decrease with increasing dEB levels, and IL-10 showed a trend to a quadratic peak at 250 mEq/kg before declining. Behavioral analysis showed a quadratic response in standing behavior, peaking at 250 mEq/kg, while position changes decreased linearly with increasing dEB. Beta diversity analysis revealed differences in unweighted UniFrac principal coordinate analysis between 230 and 290 mEq/kg dEB groups. The abundance of the actinobacteriota phylum tended to decrease linearly; however, the abundance of major phyla including the firmicutes and bacteroidota was unaffected.

**Conclusion:**

In conclusion, increasing dietary dEB from 230 to 270–290 mEq/kg with bicarbonate supplementation improved feed intake and piglet weight at weaning, suggesting that a dEB around 270 mEq/kg is optimal for supporting sow reproductive performance under heat stress.

## INTRODUCTION

Heat stress is one of the most critical challenges in modern swine production during summer when high ambient temperatures and humidity levels significantly compromise animal welfare, reproductive efficiency, and overall productivity [1,2]. Pigs are vulnerable to heat stress due to their limited capacity for evaporative cooling and relatively few sweat glands [3–5]. In swine, heat stress can lead to reduced feed intake [1,6], negative energy balance, impaired lactation performance, and increased susceptibility to oxidative stress and systemic inflammation [7,8]. These physiological changes not only impact the well-being of the sow but also have profound effects on piglet growth, survival, and overall litter performance [9]. Thus, finding effective nutritional strategies to mitigate heat stress in lactating sows is a priority for both researchers and swine producers.

Among various nutritional interventions, dietary electrolyte balance (dEB) has emerged as a promising approach to enhance thermoregulation, support metabolic homeostasis, and reduce the adverse effects of heat stress [10,11]. The concept of dEB is based on the balance between key dietary ions, sodium, potassium, and chloride, which play a fundamental role in maintaining acid-base equilibrium, osmotic balance, and cellular function [12,13]. Given that bicarbonate functions as an amphiprotic ion capable of accepting and donating protons, it can buffer both metabolic and respiratory acid-base disturbances. Therefore, substituting part of chloride with bicarbonate may more effectively stabilize blood pH under hyperventilation-induced alkalosis. Under heat stress conditions, pigs experience excessive respiratory alkalosis due to increased respiration rates [14,15], which leads to alterations in blood pH and electrolyte imbalances [16,17]. By optimizing dEB, it is possible to counteract these physiological disturbances, thereby improving the animal ability to cope with heat stress [18]. Previous studies have demonstrated that increasing dEB in the diets of pigs can enhance feed intake and overall performance [13,19]. There are several reports evaluating the dEB requirement under the normal environment; however, the optimal dEB level for sows under heat stress conditions is rarely studied [19,20]. Furthermore, the impact of dEB on immune function, oxidative stress markers, and gut integrity in heat-stressed lactating sows has not been fully elucidated. To test these hypotheses, the present study was conducted to evaluate the effects of different dEB levels on lactating sow performance, thermoregulatory responses, inflammatory markers, oxidative stress, gut integrity, and intestinal microbiota diversity during heat stress.

## MATERIALS AND METHODS

### Experimental design and management

This study was carried out during the peak heat stress period of July and August 2024. The management protocol was according to the methodologies outlined by Moturi et al [21]. Briefly, on gestation day 112, sows were transferred to farrowing crates (2.14×2.15 m), each equipped with a feeder and nipple drinkers providing uninterrupted water access. To ensure piglet comfort, heating pads were installed on both sides of the crates and maintained at 36°C. Environmental conditions, including temperature and humidity, were continuously recorded at 5-minute intervals using Tenmars temperature-humidity data loggers (TM-305U; Tenmars Electronics) placed at sow head level. These devices recorded temperature with a precision of ±0.5°C (resolution: 0.02°C) and humidity with an accuracy of ±3.4% (resolution: 0.2%). The temperature-humidity index (THI) was calculated using the equation: THI = Temperature−(0.55–[0.0055×Humidity])×(Temperature−14.5). To assess sow thermoregulatory responses, respiratory rates were recorded daily at 1,300 by counting flank movements for 60 seconds, following the approach described by Nejad and Sung [22]. A total of 40 multiparous sows (Landrace×Yorkshire; average initial body weight: 209.6±15.7 kg) were selected on gestation day 112 and divided into two parity groups (parity three and four; 20 sows per group). The sows were then randomly assigned to one of four dEB treatments: 230 mEq/kg (sodium chloride, 0.47%), 250 mEq/kg (sodium chloride 0.34%+sodium bicarbonate 0.14%+potassium bicarbonate 0.05%), 270 mEq/kg (sodium chloride 0.25%+sodium bicarbonate 0.26%+potassium bicarbonate 0.09%), and 290 mEq/kg (sodium bicarbonate 0.52%+potassium bicarbonate 0.13%). Diets were formulated according to NRC [23] recommendations, containing 13.81 MJ/kg metabolizable energy, 18% crude protein, and 0.88% standardized ileal digestible lysine. Feed intake was monitored daily by recording feed refusals. From the first day postpartum, sows were gradually offered increasing amounts of feed, starting with 1 kg and increasing by 1 kg/day until reaching 2 kg plus 0.6 kg per piglet by lactation day 7.

### Sow body weight and litter performance

Sow BW was recorded at three time points: gestation day 112 (pre-farrowing), 24 hours post-partum, and weaning (day 24 post-farrowing), following the methodology established by Rudolph et al [6]. Additionally, sow feed intake (ADFI) and weaning-to-estrus interval (days) were monitored. Backfat thickness was measured at the 10th rib at the same time points using an ultrasound scanner (Agroscan A16; Angoulême). Changes in backfat thickness were calculated as the difference between these measured values. Litter performance metrics, including total piglets born, live-born piglets, birth weight, weaning weight, and the number of piglets weaned, were documented.

### Hair cortisol analysis

Hair cortisol concentrations were analyzed following the method described by Nejad et al [24]. The foreheads of sows were shaved on gestation day 112, and the regrown hairs were shaved again on day 21 of lactation and wrapped in aluminum foil, and stored in polypropylene tubes. To eliminate surface contaminants, samples were washed three times with 5 mL of isopropyl alcohol and allowed to air-dry at 23±1.5°C for seven days. Cortisol was extracted using methanol and analyzed using a commercial ELISA kit (Cayman Chemical). The intra-assay and inter-assay coefficients of variation (CVs) were 8.32% and 9.69%, respectively.

### Acid-base indicators

The acid-base balance of the sows was assessed using venous blood collected from the ear vein into 3-mL non-heparinized vacuum tubes. Samples were analyzed within 10 minutes of collection for pH, sodium, potassium, chloride, bicarbonate, base excess, partial pressure of carbon dioxide (pCO_2_), and partial pressure of oxygen (pO_2_) using an i-STAT portable clinical analyzer equipped with EC8+ cartridges (i-STAT).

### Cytokines, antioxidant capacity, and gut integrity factors in serum

Blood samples (10 mL) were collected from the ear vein of all sows on lactation day 24. Serum was separated by transferring blood into untreated vacuum tubes, allowing clot formation at room temperature (25°C), and centrifuging at 2,500×g for 10 minutes. The serum samples were immediately stored at −20°C for subsequent biochemical analyses. The MyBioSource kit (MyBioSource) was used to evaluate concentrations of interleukin (IL)-10 (Cat# MBS2513043), IL-1β (Cat# MBS700738), tumor necrosis factor-α (TNF-α; Cat# MBS2019932), zonulin (Cat# MBS2607498), occludin (Cat# MBS740246), and total antioxidant capacity (TAC; Cat# MBS2611923) using commercial ELISA kits following the manufacturer’s instructions. Standard curves were generated for cytokine quantification, and all assays were performed in duplicate to ensure accuracy. Superoxide dismutase (SOD; Cat# MBS265304) activity in serum was assessed using a nitroblue tetrazolium-based competitive assay in conjunction with the xanthine-xanthine oxidase system, and SOD activity was expressed as μg/L. Lipid peroxidation levels were quantified by measuring malondialdehyde (MDA; Cat# MBS742540) concentrations using the thiobarbituric acid reaction method, with absorbance readings taken at 532 nm.

### Sow behavior

The Geovision GV-1240 video capture combo card (Geovision) was utilized to capture and record sow behavior during lactation. Real-time observation of these behaviors was carried out using EZViewlog (Geovision). From d 3 to 21 of lactation, the behaviors of the sows were continuously recorded for 8 h (09:00 to 17:00) each day. The behaviors, including drinking, standing, lying, sitting, feeding, nursing, and position change were recorded.

### DNA extraction and 16S rRNA amplification and sequencing

Fecal samples were collected from all sows on lactation day 24 via rectal stimulation and immediately stored at −20°C in sterile 50 mL conical tubes. Genomic DNA was extracted from 250 μL of fecal sample per subject using the QIAamp PowerFecal Pro DNA Kit (Qiagen), following the manufacturer protocol to optimize yield and minimize contamination. Extracted DNA samples were stored at −20°C until subsequent processing. The V3-V4 hypervariable regions of the bacterial 16S rRNA gene were amplified and prepared for sequencing following the standard Illumina 16S metagenomic library preparation protocol (Illumina; Part No. 15044223 Rev. B). Amplicon libraries were normalized to equimolar concentrations, pooled, and sequenced using the Illumina MiSeq platform. Sequencing reads were quality-checked, trimmed, and de-multiplexed using custom Perl scripts to reduce sequencing artifacts. Data were processed using the QIIME2 pipeline (ver. 2023.7) to evaluate microbial diversity and richness. Sequences were classified into Amplicon Sequence Variants (ASVs) based on the SILVA 138-99 reference database, with each ASV treated as a unique feature. To account for sequencing depth variability, ASV counts were rarefied to 18,911 reads per sample. Principal coordinate analysis (PCoA) based on UniFrac distance metrics was conducted, and visualizations were generated using EMPeror software.

### Statistical analyses

Statistical analyses were performed using the General Linear Model (GLM) procedure in SAS software (ver. 9.4; SAS Institute) for parametric variables, including reproductive performance, rectal temperature, respiratory rate, hair cortisol, electrolyte balance, cytokines, antioxidant capacity, and gut integrity markers. For nonparametric data, the Kruskal–Wallis test was used. Bonferroni corrections were applied to adjust for multiple comparisons and minimize type I errors. For microbial community analysis, the relative abundance of ASVs and diversity indices were statistically examined using STAMP software (ver. 2.1.3, https://beikolab.cs.dal.ca/software/STAMP). PCoA based on Bray–Curtis distance matrices was applied to evaluate group-wise β-diversity. Structural differences in microbial communities were analyzed via permutational multivariate analysis of variance (PERMANOVA). Taxonomic differences at various levels were assessed using the Kruskal–Wallis H-test and illustrated through graphical methods.

## RESULTS

### Sow performance

Dietary dEB levels during heat stress had no effect on sow BW or backfat thickness at 24 hours postpartum or at weaning (Table 1). However, ADFI during lactation exhibited a linear increase with rising dEB levels. Farrowing duration and weaning-to-estrus interval remained unaffected by dietary treatments. Similarly, dEB levels did not influence reproductive outcomes, including the total number of piglets born, born alive, weaned, piglet survivability, litter weight at birth, or litter weight at weaning (Table 2). While piglet birth weight showed no difference across treatments, piglet weaning weight exhibited a linear increasing trend with higher dEB levels.

### Blood electrolyte balance

The blood pH was linearly decreased with increasing dEB levels (Table 3). The concentration of sodium, potassium, chloride, and bicarbonate, base excess, carbon dioxide, and oxygen in the blood remained unaffected by dietary treatments.

### Heat stress factors

**Figure 1:**
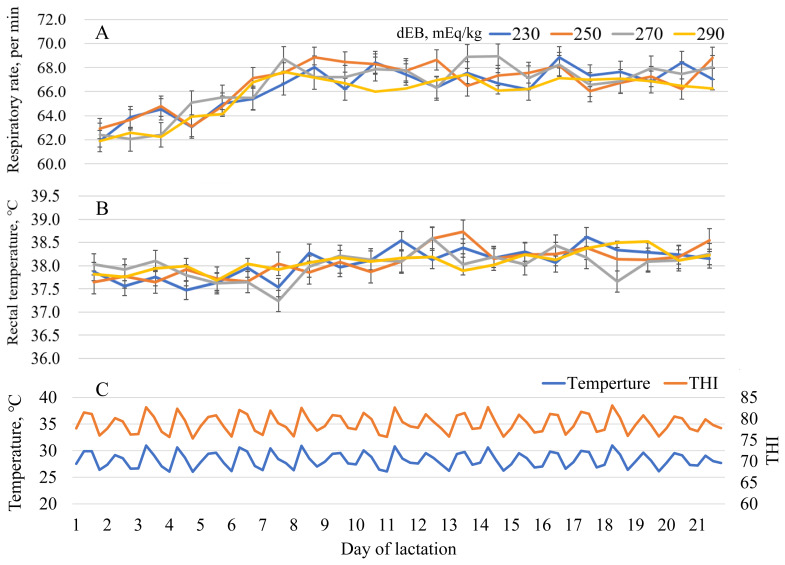
Ambient temperature and temperature-humidity index (THI) during lactation. Daily ambient temperature (blue line) and THI values (orange line) were recorded throughout the experimental period. Data were collected every 5 minutes using a digital data logger (A). Mean rectal temperatures (±SEM) were measured during lactation under heat stress across 230, 250, 270, and 290 mEq/kg dietary electrolyte balance (dEB) treatments (B). No differences were observed between treatments. Respiratory rates (±SD) were measured daily by counting flank movements for 60 seconds (C). No differences were observed among treatments. SEM, standard error of the means; SD, standard deviation.

**Figure 2:**
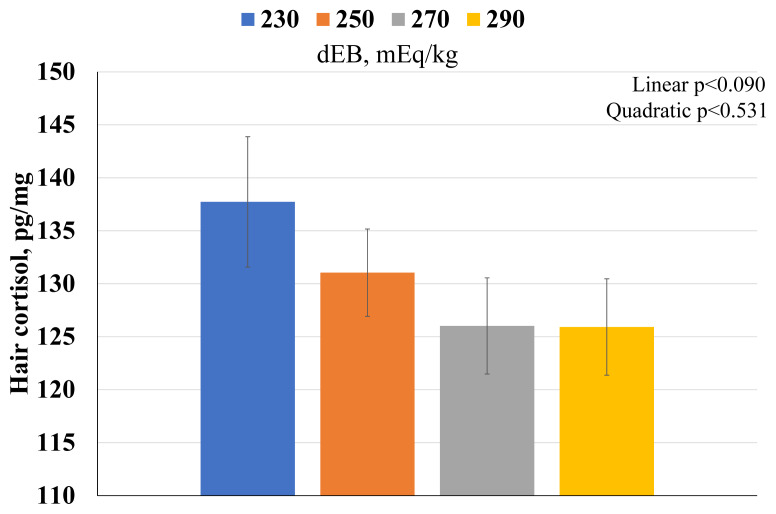
The effects of dietary electrolyte balance (dEB) on hair cortisol in lactating sows under heat stress.

The ambient temperature during the experiment ranged from 26.0°C to 31.1°C, with an average of 28.8°C (Figure 1A). No differences were observed in rectal temperature (Figure 1B) or respiratory rate (Figure 1C) among sows throughout the lactation period. Hair cortisol content showed a linear decreasing trend as dietary dEB levels increased (Figure 2).

### Inflammatory cytokine, antioxidant capacity, and gut integrity

Blood concentrations of TNF-α and IL-10 were not affected by dietary treatments (Table 4). However, IL-10 tended to peak at a dEB level of 250 mEq/kg before declining. Additionally, IL-1β showed a decreasing trend with increasing dEB levels. No differences were observed in antioxidant capacity, including TAC, SOD, and MDA concentrations (Table 4). Moreover, biomarkers of gut integrity, zonulin and occludin, were unaffected by dietary dEB levels.

### Behavior

Behavioral patterns, including drinking, standing, lying, sitting, feeding, and nursing, did not exhibit any linear responses to dietary dEB levels (Table 5). However, standing behavior followed a quadratic response, peaking at a dEB level of 250 mEq/kg before declining. Additionally, the number of position changes showed a linear decrease with increasing dEB levels.

### Variation of gut microbiota diversity

**Figure 3:**
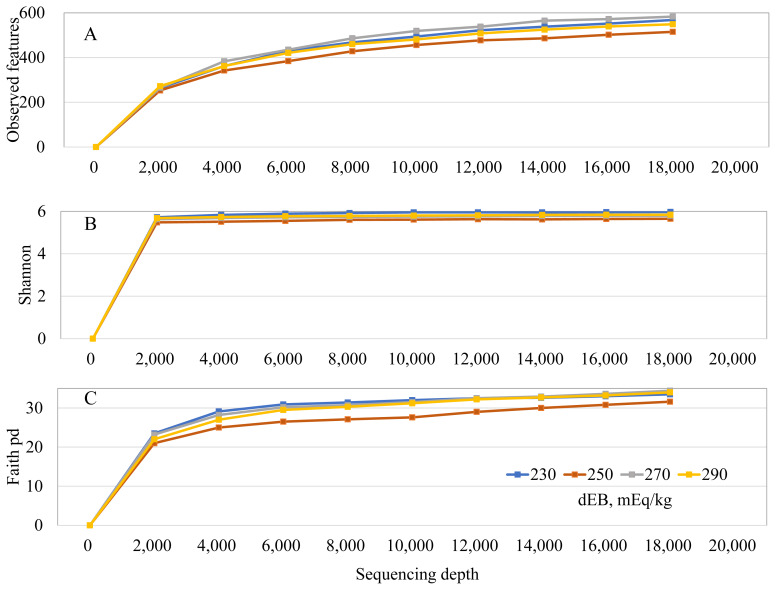
Alpha diversity of sow fecal microbiota across dietary treatments. Microbial diversity was assessed using (A) observed features, (B) phylogenetic diversity, and (C) Shannon index. Data are presented as rarefaction curves based on sequencing depth. dEB, dietary electrolyte balance.

**Figure 4:**
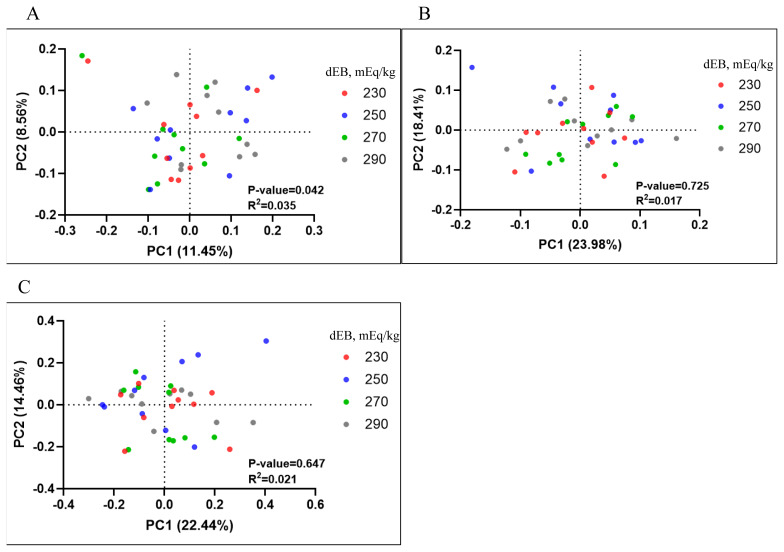
Beta diversity of sow fecal microbiota under different dietary electrolyte balance (dEB) levels. Principal coordinate analysis (PCoA) was used to visualize differences in microbial community structure based on (A) unweighted UniFrac, (B) weighted UniFrac, and (C) Bray-Curtis distances. A higher unweighted UniFrac PCoA was shown in sows fed 230 and 270 dEB levels compared with 290 mEq/kg.

**Figure 5:**
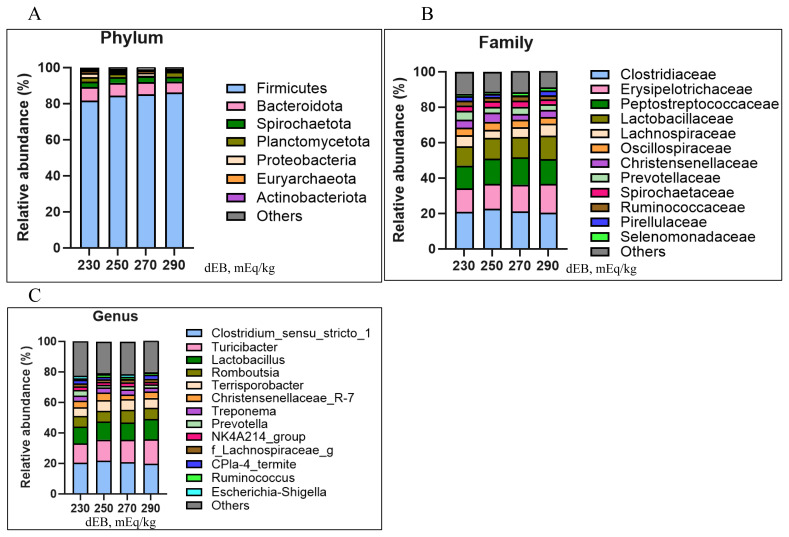
The effects of 230, 250, 270, and 290 mEq/kg dietary electrolyte balance (dEB) on lactating sows fecal bacterial community structure at the (A) phylum level, (B) family level, and (C) genus level.

The total observed features (Figure 3A), Shannon-Wiener index (Figure 3B), and phylogenetic diversity (Figure 3C) were not affected by dietary dEB levels. Beta diversity analysis revealed differences in unweighted UniFrac PCoA (Figure 4A) in sows fed 230 and 270 dEB levels compared with 290 mEq/kg, while weighted UniFrac PCoA (Figure 4B) and Bray-Curtis PCoA (Figure 4C) showed no differences among treatments. As illustrated in Figure 5A, the abundance of the Actinobacteriota phylum tended to decrease with increasing dEB levels. At the family level (Figure 5B) and genus level (Figure 5C), the relative abundances were not affected by dEB levels.

## DISCUSSION

The observed linear increase in ADFI with increasing dietary dEB suggests that higher dEB levels positively influenced nutrient intake, and partially improved systemic buffering capacity. A previous study reported that dEB values ranging from 166 to 250 mEq/kg improved BW gain, ADFI, digestibility of dry matter, and nitrogen in weaned pigs in a non-stress environment [16]. Respiratory alkalosis in lactating sows can arise from heat stress-induced hyperventilation, which leads to subsequent renal compensation [11,15,18]. The provision of alkalizing cations such as sodium and potassium can counteract this effect by enhancing bicarbonate retention, improving intracellular pH regulation, and reducing the metabolic cost of acid excretion [12,16,20]. Additionally, sodium and potassium play critical roles in extracellular fluid volume expansion to improve peripheral circulation, nutrient absorption, and feed intake [13,25]. The tendency for increased piglet weaning weight with higher dEB suggests that improved maternal nutrient intake led to enhanced milk yield and nutrient transfer. Electrolyte balance is directly associated with mammary gland function, as sodium and potassium are essential for milk secretion and osmotic balance to regulate the alveolar-to-blood gradient necessary for efficient milk ejection [10,26]. Potassium plays an important role in maintaining mammary epithelial cell integrity and secretory activity [10]. Thus, dietary electrolyte optimization may indirectly support lactation performance by improving secretory activity.

Blood acid-base balance is directly influenced by dEB, as dietary cations (sodium and potassium) and anions (chloride and sulphate) modulate systemic pH via renal and respiratory mechanisms [5,12,13]. The observed linear decline in blood pH with increasing dEB aligns with previous findings where high dEB diets reduced systemic alkalosis by promoting renal bicarbonate excretion [15,17]. Higher dietary bicarbonate intake slightly increased systemic buffering and renal regulation of acid-base equilibrium. Concurrently, increased potassium intake activates renal hydrogen-potassium ATPase, promoting hydrogen retention and mild bicarbonate excretion, explaining the observed slight decline in pH without metabolic disturbance. These mechanisms indicate adaptive acid-base regulation rather than imbalance.

Heat stress impairs thermoregulation by disrupting neuroendocrine signaling, increasing cortisol secretion, and affecting systemic acid-base status [3,9,18]. In this study, neither rectal temperature nor respiratory rate was affected by dietary dEB. However, hair cortisol levels exhibited a decreasing trend with increasing dEB, indicating a trend in the reduction of chronic stress. This effect may be attributed to improved electrolyte homeostasis by mitigating hypothalamic-pituitary-adrenal axis activation and reducing heat-induced oxidative stress.

Inflammation and immune responses are closely linked to electrolyte homeostasis, particularly through their effects on cytokine regulation and oxidative stress pathways. The observed tendency for reduced IL-1β with increasing dEB suggests the dampening of systemic inflammation under heat stress conditions. IL-1β is a pro-inflammatory cytokine involved in the acute-phase immune response and plays a crucial role in heat stress-induced immune dysregulation [4,14]. Heat stress induces oxidative stress and epithelial barrier dysfunction, endotoxin translocation, and increased IL-1β levels [8,14,27]. An optimal dEB may reduce IL-1β production by influencing intracellular signaling and immune regulation. Potassium is a critical regulator of NLRP3 inflammasome activation, which is responsible for IL-1β secretion [28,29]. Lower intracellular potassium levels are known to trigger inflammasome activation, exacerbating inflammatory responses [29]. Additionally, dietary sodium and bicarbonate contribute to systemic buffering capacity to reduce metabolic acidosis, and enhance inflammatory signaling via the NF-κB pathway [11]. NF-κB is a transcription factor that promotes pro-inflammatory cytokine expression such as IL-1β during heat stress [7,8,14]. By improving acid-base homeostasis, a higher dEB helps attenuate NF-κB activation, leading to lower IL-1β production. Deng et al [19] reported that increasing the dEB to 400 mEq/kg in weanling pig diets reduced the concentration of TNF-α, IFN-γ, and IL-1β in blood. Furthermore, electrolyte balance plays a role in maintaining intestinal integrity. Heat stress can disrupt tight junction proteins, increasing gut permeability and allowing lipopolysaccharides to enter circulation, further driving systemic inflammation [4,30]. Electrolytes such as potassium and sodium are crucial for tight junction function, and their optimal supply has been associated with improved gut barrier integrity [17]. This suggests that higher dEB may contribute to lower IL-1β levels by limiting endotoxin-induced immune activation. However, the lack of significant changes in antioxidant capacity, including TAC, SOD, and MDA, suggests that dietary dEB modifications did not substantially influence oxidative stress. This indicates that the sow’s antioxidant defense mechanisms were sufficient to counteract oxidative challenges under the tested conditions [1,11]. Additionally, markers of gut barrier integrity, zonulin, and occludin, remained unchanged, suggesting that dietary dEB variations did not compromise intestinal permeability, which is in agreement with Deng et al [19] who reported that increasing the dEB values did not improve occludens-1 and claudin-1in weaned piglets.

Behavioral changes in sows under heat stress can serve as indicators of thermal discomfort and physiological adaptation [3,6]. The number of position changes decreased linearly with increasing dEB. This suggests that moderate increases in dEB may have transiently influenced activity levels, possibly by enhancing electrolyte balance and neuromuscular function [31]. Higher dEB levels may also improve muscle relaxation through increased potassium availability, which is essential for maintaining resting membrane and neuromuscular excitability [17].

Dietary dEB did not change alpha diversity and the relative abundance of dominant phyla including the Firmicutes and Bacteroidota. By contrast, unweighted UniFrac showed significant separation between the 290 vs. 230 mEq/kg and 290 vs. 270 mEq/kg groups. This profile shift indicates a change in presence or absence of low-abundance taxa without a change in the abundance of dominant taxa. Unweighted UniFrac is driven by membership, whereas weighted metrics are driven by relative abundance; therefore, the data support a membership-level shift confined to rare taxa. Our findings suggest that the moderate reduction of chloride accompanied by moderate bicarbonate inclusion (chloride, 0.25%; bicarbonate, 0.35%) achieved similar performance to 290 mEq/kg bicarbonate inclusion. This indicates that partial replacement of chloride rather than complete substitution optimizes acid-base status without inducing excessive alkalinity. Increasing luminal pH and stabilizing the mucus layer reduce the niche for acid-tolerant and mucus-dependent low-abundance taxa, producing presence or absence differences without shifting dominant groups [32]. In pigs under heat stress, epithelial barrier weakness and ion-transport disturbances are well documented [33]. Decreasing the chloride load can decrease epithelial stress and constrain dysbiotic taxa to below-detection levels, again yielding unweighted separation [32]. In our data, Actinobacteriota tended to decrease as dEB increased, consistent with the higher luminal pH mechanism noted above and prior reports linking Actinobacteria prevalence to lower-pH niches [34]. Therefore, increasing dEB by decreasing chloride produced significant community structure changes while preserving overall diversity and dominant taxa.

## CONCLUSION

Increasing dEB from 230 to 270–290 mEq/kg by higher bicarbonate provision improved feed intake, piglet weaning weight, and inflammation resilience, suggesting that a dEB around 270 mEq/kg is optimal for supporting sow reproductive performance under heat stress. The study demonstrates that the moderate bicarbonate inclusion effectively supports reproductive performance by optimizing acid-base equilibrium.

## Figures and Tables

**Table 1:** The effects of dietary electrolyte balance on sow performance in lactating sows under heat stress

Items	dEB (mEq/kg)				SEM	Linear	Quadratic

	230	250	270	290			
BW (kg)							
Gestation (d 112)	234.5	237.3	240.0	234.2	5.18	0.908	0.249
24 h postpartum	215.3	216.4	222.6	216.3	5.30	0.579	0.336
Weaning^1)^	198.6	199.9	206.5	200.2	5.03	0.478	0.291
Loss during lactation	16.70	16.49	16.05	16.17	0.89	0.472	0.798
BF (mm)							
Gestation (d 112)	21.14	21.63	22.03	21.53	0.39	0.204	0.526
24 h postpartum	21.12	21.49	21.79	21.32	0.37	0.349	0.257
Weaning	18.08	18.46	18.85	18.33	0.38	0.357	0.106
Loss during lactation	3.04	3.03	2.94	2.99	0.16	0.652	0.778
ADFI (kg/d)							
During lactation	5.52	5.60	5.72	5.70	0.07	0.009	0.313
Farrowing duration (h)	4.46	4.61	4.62	4.46	0.14	0.965	0.113
WEI (d)	5.40	5.40	5.11	5.20	0.40	0.485	0.862

1) Day 24 post-farrowing.

dEB, dietary electrolyte balance; SEM, standard error of the means; BW, body weight; BF, back fat; ADFI, average daily feed intake; WEI, wean to estrus interval.

**Table 2:** The effects of dietary electrolyte balance on litter performance in lactating sows under heat stress

Items	dEB (mEq/kg)				SEM	Linear	Quadratic

	230	250	270	290			
Litter size (n)							
Total born	12.9	13.0	12.8	12.7	0.56	0.655	0.802
Born alive	11.7	11.5	11.1	11.2	0.42	0.157	0.613
Weaned^1)^	10.5	10.1	10.0	10.3	0.44	0.621	0.272
Survivability of piglets (%)	89.79	88.17	90.20	91.83	2.94	0.387	0.439
Litter weight (kg)							
At birth	15.54	15.17	14.67	14.69	0.50	0.783	0.574
At weaning	58.88	56.84	57.82	59.66	1.91	0.653	0.243
Piglet weight (kg)							
At birth	1.33	1.32	1.32	1.31	0.03	0.626	0.980
At weaning	5.61	5.63	5.79	5.80	0.13	0.003	0.863

1) Day 24 post-farrowing.

dEB, dietary electrolyte balance; SEM, standard error of the means.

**Table 3:** The effects of dietary electrolyte balance on blood ion and gas in lactating sows under heat stress

Items	dEB (mEq/kg)				SEM	Linear	Quadratic

	230	250	270	290			
pH	7.49	7.48	7.44	7.41	0.02	<0.001	0.731
Sodium (mmol/L)	140.6	139	140.4	136.6	2.66	0.215	0.562
Potassium (mmol/L)	6.51	6.42	6.51	6.70	0.23	0.342	0.362
Chloride (mmol/L)	173.1	165.8	163.5	156.4	20.84	0.432	0.995
Bicarbonate (mmol/L)	28.9	28.5	29.0	32.2	1.96	0.103	0.203
BE (mmol/L)	7.40	7.09	8.11	6.90	0.52	0.764	0.231
pCO_2_ (kPa)	5.82	5.92	6.21	6.20	0.28	0.122	0.791
pO_2_ (kPa)	5.78	5.68	5.41	5.27	0.86	0.740	0.777

dEB, dietary electrolyte balance; SEM, standard error of the means; BE, blood electrolyte.

**Table 4:** The effects of dietary electrolyte balance on biomarkers of inflammation, antioxidant capacity, and gut integrity in lactating sows under heat stress

Items	dEB (mEq/kg)				SEM	Linear	Quadratic

	230	250	270	290			
Inflammatory cytokine							
TNF-α (ng/L)	128.1	126.7	124.4	122.4	5.08	0.233	0.939
IL-10 (ng/L)	64.33	66.72	64.68	62.10	1.86	0.146	0.066
IL-1β (ng/L)	55.51	55.26	53.95	51.78	2.12	0.071	0.525
Antioxidant capacity							
TAC (mmol/L)	0.47	0.53	0.56	0.42	0.11	0.740	0.199
SOD (μg/L)	32.75	32.37	32.03	32.15	1.49	0.650	0.813
MDA (μmol/L)	1.56	1.47	1.41	1.56	0.12	0.881	0.169
Gut integrity							
Zonulin (μg/L)	33.39	31.25	33.04	32.31	1.64	0.783	0.546
Occludin (μg/L)	4.96	5.63	5.60	5.03	0.72	0.930	0.228

dEB, dietary electrolyte balance; SEM, standard error of the means; TNF-α, tumor necrosis factor-α; IL, interleukin; TAC, total antioxidant capacity; SOD, superoxide dismutase; MDA, malondialdehyde.

**Table 5:** The effects of dietary electrolyte balance on behavior in lactating sows under heat stress

Items	dEB (mEq/kg)				SEM	Linear	Quadratic

	230	250	270	290			
Drinking (%)	6.61	6.42	6.82	6.65	0.58	0.775	0.988
Standing (%)	9.46	10.14	9.81	8.54	0.66	0.148	0.043
Lying (%)	78.14	78.73	79.33	79.89	1.34	0.178	0.984
Sitting (%)	10.03	10.03	10.43	10.54	0.94	0.521	0.934
Feeding (%)	0.47	0.54	0.56	0.50	0.13	0.818	0.494
Nursing (%)	1.52	1.56	1.52	1.51	0.12	0.850	0.726
Position change (n)	6.39	6.30	5.36	5.28	0.42	0.003	0.984

dEB, dietary electrolyte balance; SEM, standard error of the means.

## Data Availability

Upon reasonable request, the datasets of this study can be available from the corresponding author.
